# What are the core predictors of ‘hassles’ among patients with multimorbidity in primary care? A cross sectional study

**DOI:** 10.1186/s12913-015-0927-8

**Published:** 2015-07-03

**Authors:** Charles Adeniji, Cassandra Kenning, Peter A. Coventry, Peter Bower

**Affiliations:** *NIHR School for Primary Care Research, Centre for Primary Care, Manchester Academic Health Science Centre (MAHSC), University of Manchester, Williamson Building, Manchester, M13 9PL UK; NIHR Collaboration for Applied Health Research and Care, Greater Manchester (GM-CLAHRC), Manchester Academic Health Science Centre (MAHSC), University of Manchester, Williamson Building, Manchester, M13 9PL UK; NIHR Greater Manchester Primary Care Patient Safety Translational Research Centre, Centre for Primary Care, Manchester Academic Health Science Centre (MAHSC), University of Manchester, Manchester, UK; NIHR Greater Manchester Primary Care Patient Safety Translational Research Centre, Centre for Primary Care, Manchester Academic Health Science Centre (MAHSC), University of Manchester, Manchester, UK

## Abstract

**Background:**

A limitation of service delivery in primary care in the United Kingdom is that services are often organised to manage discrete long-term conditions, using guidelines related to single conditions, and managed in clinics organised around single conditions. However, many older patients have more than one condition (so called multimorbidity). Qualitative research suggests that these patients experience ‘hassles’ in their care, including multiple appointments, poor co-ordination, and conflicting recommendations. However, there is limited quantitative evidence on the ‘hassles’ that patients with multimorbidity experience, or factors predicting ‘hassles’ in patients with multimorbidity.

**Methods:**

We conducted a cross sectional study, mailing questionnaires to 1460 patients with multimorbidity identified from the disease registers of four general practices in the UK. Patients were asked to complete a range of self-report measures including measures of multimorbidity, measures of their experience of multimorbidity and service delivery. Data were analysed using regression modelling to assess the factors predicting ‘hassles’ in patients with multimorbidity.

**Results:**

In total 33 % (*n* = 486) of patients responded to the baseline survey. The ‘hassles’ most often reported by patients related to lack of information about conditions and treatment options, poor communication among health professionals, and poor access to specialist care. There was a significant relationship between numbers of conditions, and reports of ‘hassles’. In multivariate analysis, 5 variables predicted more ‘hassles’: more long-term conditions, symptoms of anxiety and depression, younger age, being in paid employment, and not having a discussion with their GP in the last 12 months.

**Conclusion:**

Hassles are frequently reported by patients with multimorbidity in primary care. A priority for future research should be on the development of new models of care that better cater for these patients. This research highlights core hassles that need to be addressed, and the patient groups that are most at risk, which may aid in the design of these new models.

## Background

It is increasingly recognised that multimorbidity (defined as the presence of more than one long term condition) poses a challenge to health care systems based around the management of single long term conditions [1].

Patient experience and satisfaction with services remains an essential focus of current policy [2–4]. Previous research suggests that patients with multimorbidity are concerned with the need to go to multiple appointments, confused about who is caring for them, report inadequate information about their conditions and face problems communicating with their clinicians concerning their care [5–9]. This suggests that services need to place a greater focus on the experience of the patient with multimorbidity [10, 11].

Currently, there is little quantitative work on the impact of multimorbidity on patient experience of ‘hassles’ with health service delivery. This is due in part to a lack of consensus about definitions and measurement, although models are emerging [12–14].

Parchman and colleagues defined health care ‘hassles’ as ‘difficulties that patients experience during their encounters with the health care system’ [15]. The authors developed a scale (see Appendix 1) to assess ‘hassles’ and conducted a cross sectional survey involving 720 patients with single conditions or multimorbidity, randomly selected from an outpatient clinic in the US. The eligible patients were mailed questionnaires containing questions about their demographics, their needs, and current ‘hassles’. By conducting regression analyses modelling the relationship between primary care attributes and ‘hassles’ scales while controlling for patients characteristics, they reported that patients with multimorbidity experienced a higher level of ‘hassles’ than those with a single condition.

Boyd and colleagues explored the frequency and magnitude of ‘health care task difficulties’ (defined as ‘perceived difficulty in managing healthcare management tasks’) experienced by older adults with multimorbidity [14]. They tested the relationship between ‘health care task difficulties’, quality of illness care and health related quality of life using a longitudinal study involving 419 patients aged 65 years and above with multimorbidity. Patients with poor health and reporting poor quality of illness care also reported greater levels of ‘health care task related difficulty’ at baseline, while over time, patient activation predicted lower levels of ‘health care task related difficulty’, while poor health and poor quality of care predicted higher levels of difficulty.

As noted above, there is limited quantitative evidence about the difficulties that patients with multimorbidity face, and the types of patients who face the greatest difficulties. The finding from these two studies suggests that there is variation in the ‘hassles’ reported by the patients with multimorbidity. However, none of the currently available studies identifies the ‘hassles’ routinely reported and whether there is a relationship between level of multimorbidity and the ‘hassles’ reported by the patient with multimorbidity. In this study, we explore the experience of ‘hassles’ among patients with multimorbidity in primary care in the UK, to answer three research questions:

- What ‘hassles’ are routinely reported by patients with multimorbidity?
- Is there a relationship between level of multimorbidity and ‘hassles’ reported?
- What are the other factors that predict ‘hassles’ in patients with multimorbidity?

## Method

### Study design

This study is a cross-sectional analysis of baseline survey data in patients with multimorbidity in the UK. The full methods have been previously described as part of the ‘Optimising Treatment in Multimorbidity Management’ study (OPTIMUM) [16].

### Sample

The study was conducted in four large general practices in Greater Manchester, UK. Questionnaires were mailed to 1460 patients with multimorbidity identified from the disease registers in these general practices. Patients were identified from registers of long-term conditions, based on the presence of any combination of at least two of the following conditions: chronic obstructive pulmonary disease, coronary heart disease, diabetes, osteoarthritis, and depression. These conditions were chosen, as they were prevalent, demonstrated different characteristics (symptom profiles, impact on function) and were associated with different incentives in the UK health care system. Patients with any terminal illness, or severe and enduring mental health problems were excluded.

### Measures

We used the ‘*health care hassles*’ *scale*, *a* self-report measure which evaluates difficulties that patients experience during their encounters with the health care system [15]. Health care ‘hassles’ include 16 items in 5-point response scales (0–‘not a problem at all’ to 4–‘very big problem’) and a score range of 0–64, with a high score indicative of higher ‘hassles’.

For descriptive purposes, we grouped the ‘hassles’ into three categories. The first category relates to problems with seeking information and interacting with health care providers (7 items e.g., ‘lack of information about my medical condition.’) The second category relates to problems with taking medications (four items, e.g., ‘uncertainty about when or how to take my medications?’). The third category relates to problems with accessing health care (five items, e.g., ‘having to wait too long to find out the results of the lab tests or X rays’).

### Demographic characteristics

We measured age, gender, education, accommodation type, access to transport, and employment status. The education category distinguished patients with and without a formal qualification, while access to transport concerned those with and without access to a car or van. Accommodation was dichotomised into ‘owner-occupier’ and ‘other’ (including rented and living in care homes).

### Clinical characteristics

Patients self-reported conditions were adapted from a list of 22 as reported by Bayliss and colleagues [5]. For descriptive purpose, the frequency count on the number of conditions was categorised into three (2–5 conditions, 6–8 conditions and 9 or more) and in regression analyses, a continuous count on the number of conditions was used. Depression was excluded from this list of conditions, and we used a separate measure of depressive symptoms, ‘the Hospital Anxiety and Depression Scale’ (HADS). This consists of 7 items addressing depression, and 7 items addressing anxiety on a 4-point scale (from ‘0–3’ with the higher scores indicative of definitive symptoms of anxiety and depression) was used [17].

### Other characteristics

General health was measured using the Medical Outcomes Study Instrument, which assesses self-rated health on a 5 point scale and scores ranges from 1 ‘poor’ to 5 ‘excellent’ with high score indicative of better health [18].

Care for long-term conditions was measured by a question about whether patients have discussed any of their long term conditions and management with a doctor or nurse in the last 12 months.

### Analysis

- What hassles are routinely reported by patients with multimorbidity?
  We measured frequency of reporting of each of the ‘hassles’. Frequencies were categorised in to one of three groups: low (0–25 %), medium (26–50 %), and high (51 % or more).
- Is there an evidence of a relationship between level of multimorbidity and hassles reported?
  A comparison of the ‘hassles’ reported by patients in the three categories of condition counts (2–5 conditions, 6–8 conditions and 9 or more) was conducted, and the chi square test (*X*^*2*^) was used to evaluate for significance of the relationship.
- What are the other factors that predict ‘hassles’ in patients with multimorbidity?
  We conducted univariate and multivariate regression analyses. Univariate analyses explored the relationship between each independent variable (demographic, clinical, and other characteristics) and overall ‘hassles’. By including the significant predictors of ‘hassles’ identified in the univariate analyses in the multivariate analyses using the forward entry in multivariate regression, we assessed their relative contribution to the prediction of ‘hassles’. All analyses were conducted using SPSS (version 20.0).

### Ethic approval

Ethical approval was granted by Greater Manchester North Research Ethics Committee (11/NW/0563).

## Results

In summary, of the 1460 patients surveyed, 486 returned the survey (33 % response rate). The majority of the sample were female (52 %) and ages ranged from 31–91 years (mean 70 ± 10). The majority were from an older age group (51–70 years *n* = 191, 71+ years *n* = 203) with just 16 patients aged < =50 years. Table 1 displays the descriptive characteristics of the patients in the sample.

**Table 1:** Description of patients characteristics, *n* = 486 (Mean, SD, Range)

Characteristic	Frequency or mean (SD)
Gender	Female = 52 %
Age (Mean, SD, Range)	70 ± 10, 31–91
Education (completed school/GCSE as a minimum level of education for Y/N)	Education (Yes) = 60 %
Accommodation	Owner = 77 %, Rented = 23 %
Own transport	Cars = 68 %
Employment^a^	In paid job = 13 %
Number of conditions (Mean, Range)	7 ± 3.1, 2–20
Combined HADS score (Mean, Range, SD)	13 ± 7.8, 0–40
How long had LTC? (<5 years/>5 years)	<5 years = 15 % / > 5 years 85 %
Discussed their LTC and management with the GP in the last 12 months (Y/N)	Y = 78 %

^a^including those who are retired from service

- What ‘hassles’ are most frequently reported by patients with multimorbidity?
  ‘Hassles’ reported by more than half the patients related to lack of information about conditions, lack of information about treatment options, poor communication between doctors, and poor access to specialist care. Table 2 displays the frequency of ‘hassles’ most often reported.

**Table 2:** Frequency of ‘hassles’ most often reported by patients on the health care ‘Hassles’ scale, *n* = 486

0–25 (%)	26–50 (%)	51+ (%)
‘Uncertainty about when or how to take my medications’ (18 %)	‘Lack of information about why I need lab test or x-rays’ (31 %)	‘Lack of information about my medical condition’ (55 %)
‘Problems getting my medication refilled on time’ (23 %)	‘Disagreement between my doctors about my diagnosis or the best treatment for me’ (31 %)	‘Poor communication between different doctors or clinics’ (55 %)
‘Medical appointments that interfere with my work, family, or hobbies’ (24 %)	‘Having my concerns ignored or overlooked by my health care providers’ (36 %)	‘Lack of information about treatment options’ (60 %)
‘Lack of information about why I have been referred to a specialist (hospital doctor)’ (24 %)	‘Having to wait too long to find out the results of the lab tests or x-rays’ (42 %)	‘Having to wait a long time to get an appointment for specialists (hospital doctors)’ (60 %)
	‘Lack of time to discuss all my problems during scheduled appointment’ (43 %)	
	‘Difficulty getting questions answered or getting medical advice between scheduled appointments’ (46 %)	
	‘Side effect from my medications’ (48 %)	
	‘Lack of information about why my medication was prescribed to me’ (50 %)
- Is there a relationship between level of multimorbidity and ‘hassles’ reported?
  We found a significant relationship between each ‘hassle’, and levels of multimorbidity. Table 3 displays the analysis of the ‘hassles’ by multimorbidity category.

**Table 3:** Descriptive analysis of the health care ‘hassles’ by category and number of long-term conditions, *n* = 486

Categories	Health care ‘hassles’ items	Number (%) of patients with “hassle”			Overall (%) across all patients	*X* ^2 (a)^	p value
		2–5 co-morbidities	6–8 co-morbidities	9+ co-morbidities			
Hassle about information	Lack of information about my medical condition	69	95	98	266 (54.8 %)	15.5	0.000
	Lack of information about treatment options	76	106	103	290 (59.6 %)	13.5	0.000
	Lack of information about why I have been referred to a specialist (hospital doctor)	30	46	42	117 (24 %)	3.5	0.009
	Poor communication between different doctors or clinics	73	95	91	267 (55 %)	7.0	0.001
	Disagreement between my doctors about my diagnosis or the best treatment for me	39	55	48	149 (30.7 %)	2.4	0.015
	Lack of information about why I need lab test or x-rays	36	56	50	148 (30.6 %)	4.2	0.006
	Having my concerns ignored or overlooked by my health care providers	44	56	66	173 (35.7 %)	9.6	0.000
‘Hassles’ about medications	Lack of information about why my medication was prescribed to me	64	87	88	243 (50 %)	10.1	0.000
	Problems getting my medication refilled on time	27	47	38	114 (23.4 %)	3.0	0.012
	Uncertainty about when or how to take my medications	23	36	27	88 (18.1 %)	0.7	0.041
	Side effect from my medications	66	91	76	233 (48 %)	2.6	0.012
‘Hassles’ about care	Having to wait a long time to get an appointment for specialists (hospital doctors)	86	99	94	292 (60 %)	2.9	0.011
	Having to wait too long to find out the results of the lab tests or x-rays	52	78	67	206 (42.3 %)	4.5	0.005
	Difficulty getting questions answered or getting medical advice between scheduled appointments	58	73	83	223 (46.0 %)	11.5	0.000
	Lack of time to discuss all my problems during scheduled appointment	51	76	73	208 (42.9 %)	9.3	0.000
	Medical appointments that interfere with my work, family, or hobbies	31	44	35	115 (23.7 %)	0.6	0.039

Chi square text *X* ^2^ (a) = (linear by linear association)
- What other factors predict ‘hassles’ in patients with multimorbidity?
  Table 4 displays the regression analysis, used to explore the relationship between independent variables and the dependent variable (‘hassles’).

**Table 4:** Displays the regression analysis, used to explore the relationship between independent variables and the dependent variable (‘hassles’), *n* = 486

	Descriptive	Univariate analysis	Multivariate analysis
Independent Variable	(Mean, SD, range, or %)	Coefficient (95 % CI)	Coefficient (95 % % CI)
	Demographic		
Gender	Female = 52 %	0.021 (−0.113, 0.180)	−0.023 (−0.180, 0.107)
Age	70 years ± 10, 31–91	−0.207 (−0.023, −0.009)**	−0.102 (−0.016, 0.000)*
Education	Formal qualifications =60 %	0.012 (−0.001, 0.000)	0.046 (−0.002, 0.001)
Accommodation type	Owner = 77 %	0.120 (0.053, 0.398)*	0.069 (−0.042, 0.304)
Number of cars/Vans owned : No Car; Car	Access to cars = 68 %	0.015 (−0.134, 0.186)	0.037 (−0.100, 0.228)
Current in employment	Paid work = 13 %;	−0.131 (−0.530, 0.097)*	−0.099 (−0.464, −0.009)*
Number of conditions	7 ± 3.1, 2–20	0.219 (0.033, 0.078)**	0.167 (0.018, 0.066)**
Combined Anxiety and Depression (HADS) score	13 ± 7.8, 0–40	0.400 (0.033, 0.051)**	0.352 (0.025, 0.050)**
	Others		
Duration with long term conditions	less than 5 years = 15 %	0.002 (−0.201, 0.209)	−0.044 (−0.291, 0.093)
Overall health score	Poor = 16 %, fair = 42 %, good = 33 %, very good = 8 %, excellent = 1 %,	−0.225 (−0.289, −0.125)**	−0.023 (−0.117, 0.075)
Discuss about their LTC with their GP in the last 12 months	Yes = 78 %	−0.096 (0.367, −0.010)*	−0.095 (−0.348, −0.018)*

*p < 0.05 and**p < 0.01

The initial univariate analysis showed that there was a significant relationship between seven independent variables in the univariate analysis. These variables were younger age, living in owned accommodation, being in paid employment, higher number of conditions, presence of anxiety and depression symptoms, poorer general health, having regular discussion about long-term conditions and management with the GP within the last 12 months (Table 4).

Five of the variables remained significant when entered into the multivariate analysis. Being of younger age (standardised beta of −0.102, *t*-test = −1.990, *P* = 0.047) and being in paid employment (standardised beta of −0.099, *t*-test = −2.040, *P* = 0.042) remained associated with higher levels of ‘hassles’. Both clinical variables also remained positively associated: number of conditions (standardised beta of 0.167, *t*-test =3.383, *P* = 0.001), and presence of anxiety/ depression (standardised beta of 0.352, *t*-test =6.007, *P* = 0.000). Seeing the GP about their long-term conditions in the last 12 months was associated with reduced ‘hassles’ (standardised beta of −0.095, *t*-test = −2.176, *P* = 0.030) (Table 4).

## Discussion

### Summary

Our analysis explored the ‘hassles’ experienced by patients with multimorbidity, and examined predictors of ‘hassles’. The results show that the ‘hassles’ most frequently reported are related to lack of information about medical conditions, lack of information about treatment options, poor communication, and poor access to specialist care.

Increase in the number of conditions was associated with increase in the level of ‘hassles’ reported. Overall, five variables predicted more ‘hassles’: more long-term conditions, symptoms of anxiety and depression, younger age, being in paid employment, and not having a discussion with the GP in the last 12 months.

### Strengths and limitations of the study

The study is essentially a replication of the earlier Parchman study [15]. The health care systems in the two settings (Greater Manchester, United Kingdom and South Texas, United States) are different, as were the populations included. For example, the patients in the US study were mostly male, and significantly younger (average age 50 vs. 70 years). This suggests that patient experience of hassles may be fairly common, even in different settings, and that different health care systems face common challenges.

Concerns must be raised by the response rate. The postal methods adopted for distribution of questionnaires provided an efficient way to collect relatively large amounts of information from patients, but this may have contributed to the response rate of 33 %. This response rate is consistent with those using postal surveys with patients in primary care [19–21] and with large scale routine NHS surveys in primary care [22], but does leave the study vulnerable to bias in terms of the external validity of the results. For example, patients with more significant functional impacts of multimorbidity may have been less likely to respond. However, such bias may have less impact on examination of associations between variables, than on estimates of rates. It is also worth noting that, even if the rates of reporting of hassles were far lower in non-respondents, this would still mean that a significant proportion of patients report important problems in care, such as a ‘lack of information about my medical condition’ or ‘poor communication between different doctors or clinic’.

An implicit assumption of the current interests around ‘hassles’ is that greater numbers of long term conditions will lead to greater ‘hassles’, as patients will be required to manage more treatments, do more self-management, travel more, and receive more attention from services [11–13]. This assumption was confirmed in the present study. To recruit patients through disease registers, this study focused on 5 conditions. It is possible that certain combinations of conditions are more prone to ‘hassles’. For example, some have distinguished between ‘discordant’ and ‘concordant’ conditions [23]. This work could be usefully replicated with other patient populations. At present, no consensus exists around appropriate typologies, and the present sample size was insufficient to explore such issues in depth.

### Comparisons with other studies

This study is the first that we know of examining the predictors of ‘hassles’ in patients with multimorbidity in the United Kingdom.

These results support the findings of Parchman and colleagues, confirming greater ‘hassles’ in patients with multimorbidity (although they had compared those with single morbidity and multimorbidity) [15]. Other published studies have not explored wider predictors of ‘hassles’. However, one study by Bower and colleagues found few differences in patient experience of chronic illness care between those with single or multiple long term conditions, although the measure used was different [24].

### Implications of research for policy, clinical practice, and future research

Research evidence has shown that many older patients visit primary care with multimorbidity, and has highlighted the requirement to develop ways of improving health care services delivery to be responsive to the needs of these patients [25–28]. The present work can contribute to developing such models, by identifying the key ‘hassles’ and those patient groups who are most at risk of that experience.

It is not clear whether the associations with depression and anxiety represent reporting issues or whether there is a clinically more important effect. It is possible that high levels of ‘hassles’ cause anxiety and depression symptoms, which would be implied by some of the qualitative work in this area. [29] A recent qualitative study reported that managing multimorbidity could lead to feelings of guilt and problems in relationships with others in the social network [13]. However, it is equally true that patients with depression may report more negatively on their experiences of care [30]. Exploring the causal mechanisms will be critical in developing new models and interventions to reduce ‘hassles’, as the implications are very different.

Although older patients are more likely to report multimorbidity, ‘hassles’ are not exclusive to the older population [25]. It is noteworthy that younger age was associated with high reports of ‘hassles’. Data on patient experience suggests that older people are generally more satisfied with services and complain less [31–34]., so this may reflect a reporting issue. It is important to note that the age effect did not simply reflect greater flexibility of older, retired patients in terms of time for appointments, as the analysis also suggested that multimorbidity in the context of paid employment was independently associated with high level of ‘hassles’. In the United Kingdom, there is significant pressure for primary care practices to provide access outside normal hours [35–37].

The frequency of discussions with a GP about long-term conditions (around 80 %) confirms previous work [21] and one interpretation of the findings is that a recent discussion with the GP might be important in reducing ‘hassles’. Certainly, recent discussions have highlighted the importance of continuity of care in multimorbidity [38].

In the United Kingdom, policy innovations such as care planning [39] or Year of Care [40] have been highlighted as methods of improving care for long-term conditions. Based broadly on the Chronic Care Model [41], the goal is to better support patients through individualised assessment of behaviour, collaborative goal setting, self-management support, and proactive follow-up. At present, most care planning is around single conditions [39, 40], and there may need to be modifications to take account of multimorbidity, such as those outlined recently in the Ariadne principles [42].

There has also been recent interest in developing models of consultation in primary care that have an explicit focus on reducing ‘hassles’, in line with what has been called ‘minimally disruptive medicine’ [10]. Care planning in multimorbidity could be used to enhance such care, although there is little evidence as yet on the effectiveness and cost-effectiveness of these new models of care [43], and their evaluation remains a priority [44].

## Conclusions

Factors that predicts health care ‘hassles’ in patients with multimorbidity are higher numbers of long term conditions, current employment, and higher depression and anxiety. Hassles are reduced in when patients have discussions with the GP about long-term conditions, and in older patients. Further longitudinal modelling is required to explore the causal relationships between those factors, which can then inform interventions and changes to service delivery to support better patient experience in multimorbidity.

## Appendix: Parchmans’ hassles scale

**Table:**

Items	Health Care ‘Hassles’
1	Lack of information about my medical condition
2	Lack of information about treatment options
3	Lack of information about why my medication was prescribed to me
4	Problems getting my medication refilled on time
5	Uncertainty about when or how to take my medications
6	Side effect from my medications
7	Lack of information about why I have been referred to a specialist (hospital doctor)
8	Having to wait a long time to get an appointment for specialists (hospital doctors)
9	Poor communication between different doctors or clinics
10	Disagreement between my doctors about my diagnosis or the best treatment for me
11	Lack of information about why I need lab test or x-rays
12	Having to wait too long to find out the results of the lab tests or x-rays
13	Difficulty getting questions answered or getting medical advice between scheduled appointments
14	Lack of time to discuss all my problems during scheduled appointment
15	Having my concerns ignored or overlooked by my health care providers
16	Medical appointments that interfere with my work, family, or hobbies
